# Epstein-Barr virus positive B-cell lymphoproliferative disorder/polymorphous B-cell lymphoma of the urinary bladder: A case report with review of literature

**DOI:** 10.4103/0970-1591.45552

**Published:** 2009

**Authors:** Sandhya Sundaram, Kai Zhang

**Affiliations:** Sri Ramachandra University, Porur, Chennai India & Fellow, Weis Center for Research, USA; 1 Geisinger Medical laboratory, Danville, Pennsylvania, USA

**Keywords:** Lymphoproliferative disorder, senile and urinary bladder

## Abstract

We report an unusual case of a localized Epstein-Barr virus (EBV)-positive B cell lymphoproliferative disorder (LPD)/polymorphous B cell lymphoma of the urinary bladder in a 67 years old female patient. She had no known predisposing immunodeficiencies and presented with recent onset of hematuria. The CT and cystoscopic examination revealed a localized 2.5 cm polypoid or plaque-like mucosal mass on the right posterior and lateral wall of the bladder. The biopsy sample showed a diffuse and densely polymorphous atypical lymphoid infiltrate admixed with numerous small lymphocytes, histiocytes and occasional plasma cells and neutrophils. The large atypical cells were CD20+, CD79a+, CD30+, CD43+ and they were strongly positive for EBV by in situ hybridization using anti-EBER-1 probe. Polymerase chain reaction (PCR) for immunoglobulin heavy chain gene rearrangement study showed a clonal gene rearrangement. The findings indicated EBV+LPD of the bladder. Primary lymphoma of bladder is rare and primary EBV+LPD of the bladder has not been previously described. Potential misdiagnosis of poorly differentiated urothelial carcinoma can occur and accurate diagnosis depends on comprehensive immunohistochemical and molecular workups.

## INTRODUCTION

A significant progress has been made in understanding the pathogenesis of Epstein-Barr virus (EBV) positive lymphoproliferative disorders (LPDs) in last decade. The immunodeficiency-associated LPDs are currently classified by the World Health Organization into the following four main groups;[1] LPDs associated with primary immunodeficiency syndromes and other primary immune disorders;[2] lymphomas associated with infection by the human immunodeficiency virus (HIV);[3] post transplant LPDs (PTLDs) in patients who have received solid organ or bone marrow allograft[4] and methotrexate-associated LPDs that is seen most commonly in patients with autoimmune disease treated with methotrexate. Recently a new clinical and pathologic entity called senile EBV+ LPDs has been described in senile patients over the age of 60 years without any predisposing immunodeficiencies.[2–4] These patients are believed to be deficient in immune surveillance against oncogenic viruses, especially the EBV that induces EVB-driven lymphoproliferative disorders.

## CASE REPORT

**Figure 1:**
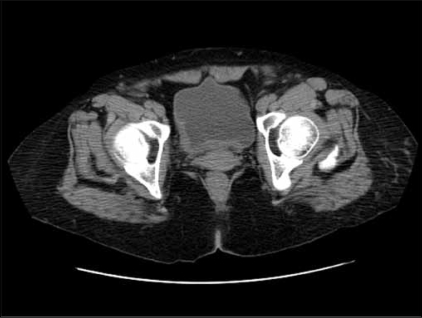
Imaging picture showing a plaque-like lesion in the bladder

A 67-year-old severely mentally retarded woman presented with recent onset of haematuria. She had past history of rheumatic heart disease, status post bioprosthetic aortic replacement as well as mitral valve replacement and tricuspid valve repair. She had a history of pulmonary hypertension related to right heart failure and was status post aortic artial septal defect repair. She was on multiple medications for medical problems. Physical examination showed no fever or lymphadenopathy. The white blood cell count was 5,500/ul with mild lymphopenia (1% lymphocytes) and mild anemia with Hb at 10.5mg/dl. The imaging [Figure 1] and cystoscopic examination revealed a localized 2.5 cm plaque-like mucosal mass on the right posterior and lateral wall of the bladder and a biopsy sample was obtained from the lesion.

The biopsy sample was fixed in 10% buffered formaldehyde and routine H&E preparation was done for histological examination. Immunohistochemistry was performed on the formalin fixed paraffin embedded tissue sections using the avidin biotin peroxidase complex method in a Dako autostainer.[5] The following antibodies from Dako were used: CD20, CD79a, CD3, CD5, CD7, CD4, CD8, CD30, CD15, MIB-1, Tdt, CD-56 CD68, S100, myeloperoxidase, ALK-1, CD117, CD35, CD1a, CD43, CD23, CD31, CD34, CK7, CK20, cytokeratin -AE1/AE3 and alpha smooth muscles actin. In situ hyvridization (ISH) for EBV was performed using anti-EBER-1 probe (Dako) according to a previously described method. Polymerase chain reaction (PCR) for B cell gene rearrangement was done using the standard method (Quest Diagnostics Incorporated) to detect the clonality of the immunoglobin heavy chain on chromosome 14.[5]

**Figure 2:**
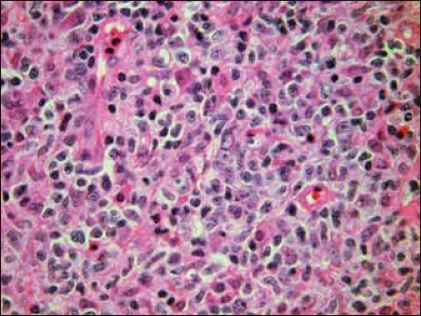
Tumour shows diffuse, large, atypical polymorphic lymphoid infiltrates (H&E, ×400)

**Figure 3:**
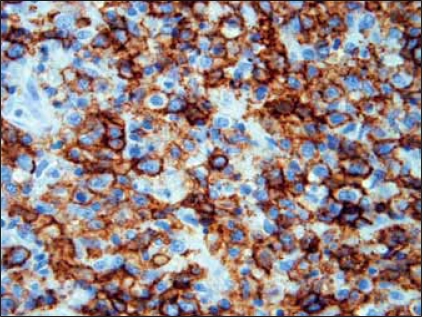
Most large, atypical lymphoid cells are CD20 positive. ×400

**Figure 4:**
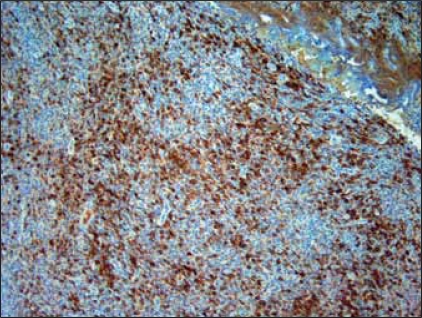
Many cells also show CD30 positivity. ×200

**Figure 5:**
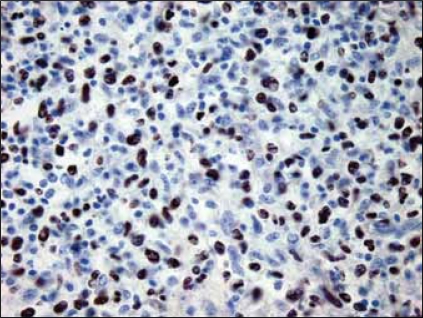
Most cells are EBV positive (anti -EBER probe), × 200

H&E sections showed a diffuse and densely atypical polymorphous mixed small and large lymphoid infiltrate involving mucosa and deep muscles of the bladder [Figure 2]. The atypical cells showed a spectrum of cell sizes and shapes, and many large atypical lymphoid cells formed small clusters and/or sheets, and some atypical cells showed plasmacytic differentiation. The background cells were composed of numerous small lymphocytes, histiocytes, occasional plasma cells and neutrophils. The large atypical cells were CD20+ [Figure 3], CD79a+, CD30+9 [Figure 4], CD43+, EMA+ (focally); but negative for CD2, CD5, CD8, CD4, CD7 and myelomonocytic markers including (myeloperoxidase) MPO, CD117, CD68 (PGM-1), as well as CD10, terminal deoxynucleotidyl transferase (Tdt) and ALK-1. The majority of atypical large lymphoid cells were strongly positive for EBV by ISH using anti-EBER-1 probe [Figure 5]. PCR for immunoglobulin heavy chain gene rearrangement study was positive.

## DISCUSSION

We report an unusual case of localized primary EBV positive LPDs of the urinary bladder, which has not been previously reported. The patient had lymphopenia that may contribute to the pathogenesis of the disorder. The overall features of the case resemble post-transplant LPDs. Morphologically the disorder may potentially be misdiagnosis as poorly differentiated urothelial carcinoma given the polymorphic atypical large cells and location of the tumor. Attention should be paid to cell composition and lack of clear evidence of epithelial or urothelial differentiation. A comprehensive immunohistochemical panel was required for precise and accurate diagnosis.

Lymphoepithelioma like carcinoma of the bladder forms a close differential diagnosis,[6] as these lesions also present with frank heamaturia and comprise of tumor cells showing a syncytial growth pattern, with poorly defined cytoplasmic borders. A dense cellular infiltrate is present, consisting of mature lymphocytes often admixed with plasma cells and histiocytes. Although lymphoepithelioma like carcinoma in nasopharyngeal carcinomas have a strong association with the EBV Lopez *et al.* failed to demonstrate the presence of EBV in any of the cases in the bladder.[7] Immunohistochemistry for cytokeratins is invaluable for distinguishing primary bladder lymphoma and LPD'S from lymphoepithelioma like bladder carcinoma.[8] Accurate diagnosis is therefore very important as it has therapeutic implications.

Recently, Oyama *et al.*[2] described 22 cases of EBV associated (EBV-positive) B cell LPDs in old patients (over 60 years with a median age of 76 year) without any predisposing immunodeficiencies presenting with predominantly with extranodal involvement (82% of the cases). Biopsied specimens contained varying numbers of centroblasts, immunoblasts, and Hodgkin and Reed-Sternberg (HRS)-like cells. The most frequent sites of extranodal involvement were skin and respiratory tracts, and the other sites included liver, spleen, tonsils, stomach, salivary glands, bone marrow, pancreas, and oral cavity. Their clinical behavior varied widely, from an indolent process with spontaneous remission in one case, to an aggressive and fatal course. The new clinical and pathologic entity was named as senile EBV+ LPDs. Subsequently a study of 76 patients with senile EBV B-cell LPD was reported by Shimoyama *et al.*[3] showed similar features as described initially. A similar case report of senile LPD occurred in nasopharynx.[4] The mounting evidence suggests that this disease represent a distinctive entity and it may be related to the immunological deterioration or senescence in the immune system that occurs during the aging process.

The patient was given planned course of palliative radiation therapy under general anesthesia in view of her inability to cooperate otherwise. She was given 800 cGy in 3 fractions thereby totaling 2400 cGy. The patient tolerated the treatment well without any obvious sequelae. She had the desired effect from treatment with resolution of hematuria. To date, she is alive without any lymphoma-related illness. Awareness of this rare entity is necessary to avoid erroneous diagnosis. The case reported here shared certain features of senile EBV positive LPDs and falls in the spectrum of this newly categorized disorder.
